# Glycoproteins of Predicted Amphibian and Reptile Lyssaviruses Can Mediate Infection of Mammalian and Reptile Cells

**DOI:** 10.3390/v13091726

**Published:** 2021-08-30

**Authors:** Martina Oberhuber, Anika Schopf, Alexandru Adrian Hennrich, Rosalía Santos-Mandujano, Anna Gesine Huhn, Stefan Seitz, Christiane Riedel, Karl-Klaus Conzelmann

**Affiliations:** 1 Max von Pettenkofer-Institute Virology & Gene Center, LMU Munich, 81377 Munich, Germany; oberhuber@genzentrum.lmu.de (M.O.); schopf@genzentrum.lmu.de (A.S.); hennrich@genzentrum.lmu.de (A.A.H.); santos-mandujano@genzentrum.lmu.de (R.S.-M.); 2 Department of Infectious Diseases, Molecular Virology, University of Heidelberg, 69120 Heidelberg, Germany; anna.huhn@path.ox.ac.uk (A.G.H.); s.seitz@dkfz-heidelberg.de (S.S.); 3 Institute of Virology, University of Veterinary Medicine Vienna, Vienna 1210, Austria; Christiane.Riedel@vetmeduni.ac.at

**Keywords:** rabies virus, zoonosis, emerging disease, anole lyssavirus, frog lyssavirus, host range, neurotropism, vaccine

## Abstract

Lyssaviruses are neurotropic rhabdoviruses thought to be restricted to mammalian hosts, and to originate from bats. The identification of lyssavirus sequences from amphibians and reptiles by metatranscriptomics thus comes as a surprise and challenges the mammalian origin of lyssaviruses. The novel sequences of the proposed American tree frog lyssavirus (ATFLV) and anole lizard lyssavirus (ALLV) reveal substantial phylogenetic distances from each other and from bat lyssaviruses, with ATFLV being the most distant. As virus isolation has not been successful yet, we have here studied the functionality of the authentic ATFLV- and ALLV-encoded glycoproteins in the context of rabies virus pseudotype particles. Cryogenic electron microscopy uncovered the incorporation of the plasmid-encoded G proteins in viral envelopes. Infection experiments revealed the infectivity of ATFLV and ALLV G-coated RABV pp for a broad spectrum of cell lines from humans, bats, and reptiles, demonstrating membrane fusion activities. As presumed, ATFLV and ALLV G RABV pp escaped neutralization by human rabies immune sera. The present findings support the existence of contagious lyssaviruses in poikilothermic animals, and reveal a broad cell tropism in vitro, similar to that of the rabies virus.

## 1. Introduction

According to textbooks, the hosts of lyssaviruses, a genus in the *Rhabdoviridae* family, are homothermic animals. Bats are the prime hosts and reservoirs of almost all of the identified 17 lyssavirus species [1] and it was thus suggested that lyssaviruses originate from bats [2,3]. The prototypical rabies virus is atypical in terms of host range, as it is endemic in both bats and terrestrial carnivores. In addition, Mokola and Ikoma lyssaviruses have been isolated exclusively from terrestrial mammals.

Phylogenetic analyses of lyssaviruses reveal a close relationship and substantial distance from other rhabdovirus genera. Members of the genus are allocated to phylogroups, primarily according to the serologic cross-reactivity of the G proteins [4,5]. Phylogroup I so far comprises 11 lyssavirus species that show cross-neutralization by the antibodies of rabies vaccines [6]. Phylogroup II includes Lagos bat lyssavirus (LBV), Mokola lyssavirus (MOKV), and Shimoni bat lyssavirus (SHIBV) [7], and the most remote phylogroup III is represented by West Caucasian bat lyssavirus (WCBV) [8], Lleida bat lyssavirus (LLEBV) [9], and Ikoma lyssavirus (IKOV) which was isolated from terrestrial mammals only [10].

Recently, novel lyssavirus sequences were identified in the neuronal tissue of frogs and lizards, by screening publicly available sequence data. Specifically, virus sequences were found in male American green treefrogs (*Dryophytes cinereus*, *Hyla cinerea*), and referred to as a frog lyssa-like virus (FLLV-1) (GenBank MK473367.1 and MK473368.1). We are here using the abbreviation ATFLV to provide space for other frog lyssaviruses to be identified. The first reptile lyssavirus sequence (GenBank BR001666.1) was identified in the tissue of a Spanish flag anole (*Anolis allogus)* and is referred to as anole lizard lyssavirus (ALLV) or anole lyssavirus-like virus (ALLV-1) [11].

The assembled amphibian and reptilian lyssavirus sequences cover almost the entirety of viral genomes, except for a few residues at the ends, allowing for the clear-cut assignment to the lyssavirus genus and demarcation from other rhabdovirus genera. They reveal typical lyssavirus features, including a genome organization with non-overlapping N, P, M, G, and L open reading frames, almost identical and complementary 3′ends, and conserved lyssavirus transcription stop/restart sequences. A phylogenetic analysis revealed that ATFLV and ALLV represent sister lineages to the mammalian lyssaviruses with which they build a monophyletic group within the *Rhabdoviridae* family [11]. Of note, the anole ALLV is genetically much closer to mammalian phylogroup III lyssaviruses than to the frog ATFLV, revealing a remarkable genetic and evolutionary distance between amphibian and reptile lyssaviruses.

Most significantly, ATFLV and ALLV lyssavirus sequences were identified in neuronal tissue samples, indicating that the tropism of the predicted viruses matches that of the neurotropic mammalian lyssaviruses. To our knowledge, none of these viruses could be isolated from infected specimens so far. Thus, to shed light on the question of whether the novel sequences reflect the existence of contagious amphibian and reptilian lyssaviruses, we expressed here the encoded glycoproteins from plasmids and pseudotyped G-deficient RABV with AFTLV and ALLV G proteins. Notably, both G proteins could readily mediate the infection of cell lines not only from reptiles, but also of cell lines from bats, rodents, and humans, including cells of neuronal origin, revealing the full functionality and RABV G-like phenotype with respect to permissive cell types.

## 2. Materials and Methods

### 2.1. Cell Culture

HEK293T (ATCC CRL-3216), VeroE6 (ATCC CRL-1586), and N2A (ATCC CCL-131) cells were maintained in DMEM medium (GIBCO) containing 10% fetal bovine serum and 1% L-glutamine (200 mM, GIBCO). SHSY-5Y (ATCC CRL-2266) cells were maintained in DMEM-F12 GlutaMAX with 10% FBS and 1% MEM non-essential amino acids solution (100X). BSR T7/5 [12] and BHK-MG-on cells [13] were grown in GMEM media containing 10% fetal bovine serum, 1% MEMs/NEAAs, and 19.5 mL tryptose phosphate broth (Thermo Fisher Scientific, Waltham, MA, USA). The expression of SAD G in BHK-MG-on cells was induced by adding 1 μM doxycycline. Bat cells MVI/it (*Myotis velifer incautus*, interscapular tumor, ATCC CRL-6012), TB1-Lu (*Tadarida brasiliensis* lung), Rhi-Lu-hACE2 (*Rhinolophus* sp., lung, stably transduced with human ACE2), and MyDauNi/2 (*Myotis daubentonii*) were kindly provided by MA Müller [14]. MVI/it and TB1-Lu were maintained in DMEM with 10% FBS and Rhi-Lu-hACE2 and MyDauNi/2 in DMEM with 10% FBS, 1% MEM non-essential amino acids solution (100×), 1% L-glutamine (200 mM), and 1% sodium pyruvate (100 mM). All mammalian cells were grown at 37 °C under 5% CO2 in the presence of 0.5% penicillin-streptomycin (10,000 U/mL, Gibco).

Reptile cells TH-1 (*Terrapene carolina* (common box turtle) heart; CCLV-RIE 1131), SKH-R (*Trachemis scripta elegans* (red-eared slider) juvenile (3–4 weeks) heart; CCLV-RIE 0483), VH2 (*Daboia russelli* (Russell’s viper) heart, adult female, CCLV-RIE 1092), and IgH-2 (*Iguana iguana* (common green iguana) heart immature male; CCLV-RIE 1217) were kindly provided by Matthias Lenk, FLI Riems. All reptile cell lines were cultured in GMEM with 10% FBS, 1% MEM non-essential amino acids solution (100×), 4% tryptose phosphate broth and 0.5% penicillin-streptomycin (10,000 U/mL).

### 2.2. Expression Plasmids and Complementation of RABV pp

Original sequence ALLV G (GenBank BR001666.1) and ATFLV G (MK473367.1) cDNAs were synthesized by BioCat, Heidelberg and cloned via *Nhe*I and *Xba*I in pcDNA3.1(+) expression plasmids. The SADΔG-eGFP replicon has been described before [15], and SADΔG-GLuc-mNeongreen was cloned by replacing the eGFP cistron with two cistrons encoding *Gaussia* luciferase and mNeongreen, respectively. The virus rescue was performed in BSR T7/5 cells transfected with viral cDNA plasmids directing T7 RNA polymerase-driven antigenome(+) RNAs along with expression plasmids encoding helper proteins N, P, and L [12,16]. Both replicons were amplified in BSR MG-on cells inducibly expressing SAD G [13].

Stocks of SAD G-complemented RABVΔG pp were used to infect a BHK cell line (BHK-EnvA^RT^) expressing the envelope glycoprotein from the avian sarcoma leukosis virus (ASLV-A) known as EnvA and containing the SAD G C-tail (EnvA^RT^) at a MOI of 1. After 6 h of incubation at 37 °C, cells were trypsinized, washed, and resuspended. The infection was controlled by eGFP fluorescence and supernatant viruses were concentrated by ultracentrifugation through a 20% sucrose cushion in an SW32 rotor at 24,000 rpm and 4 °C for 2 h, and aliquots stored at −80 °C. HEK293T-TVA cells transfected with rhabdovirus G-encoding plasmids were infected with the resulting EnvA^RT^ pp and the G-pseudotyped particles were harvested 3 and 4 days post infection from culture supernatants and concentrated by ultracentrifugation.

### 2.3. Sequence Analysis

G protein sequences were collected from Uniprot (SAD, P16288; IKOV, J7JVS8; LLEBV, A0A1I9RGZ9; WCBV, Q5VKN9; ATFLV, A0A6G5RSD8; VSV, P03522) or from NCBI GenBank (EBLV1 (EU293109.1/protein_id ABZ81165.1), MOKV (EU293117.1/ABZ81205.1), and ALLV (BR001666.1/FAA01391.1). Sequences were aligned with Clustalω using default parameters. Biochemical properties and conservation were calculated with ClustalX.

### 2.4. Virus Neutralization Assay

Inactivated serum from three rabies vaccinated volunteers with rabies antibody titers >0.5 IU/mL as determined by an accredited laboratory or control serum was 10-fold serially diluted, incubated with 600 SADΔG-GLuc-mNeongreen particles in a volume of 100 µL for 30 min, and added to the monolayers of BSR T7/5 cells in clear 96 well plates. After incubation for 3 days, aliquots of supernatants were used to determine luciferase activity (*Renilla* Luciferase Assay System, Promega) in a plate reader (Mithras LB 940, Berthold Technologies, Bad Wildbad, Germany). Dilutions of the vesicular stomatitis virus (VSV)-specific hybridoma supernatant (I1-Hybridoma, ATCC CRL-2700) were used as control for neutralization of VSV G pp.

### 2.5. Cryo-Electron Microscopy

Concentrated preparations of RABV pp were added to glow discharged Quantifoil 200 mesh 2/1 holy carbon copper grids in the presence of Aurion protein A 10 nm gold beads. Vitrification was performed with a manual plunging unit. Grids were analyzed in a Glacios cryo electron microscope (Thermo Fisher Scientific, Waltham, MA, USA) operated at 200 kV, and images were acquired with a Falcon 2 direct electron detector (Thermo Fisher).

## 3. Results

### 3.1. Organization of G Proteins from Poikilothermic Lyssaviruses

The entry of lyssaviruses into host cells is mediated by their single type I transmembrane glycoprotein G. An N-terminal signal peptide directs synthesis of the protein into the ER, and after cleavage of the signal peptide, the nascent protein is anchored in the membrane by a single-pass alpha-helical transmembrane domain. A short cytoplasmic tail (CT) is involved in association with matrix protein (M)-coated viral ribonucleoproteins [17] at the site of virus envelope assembly and budding [18,19]. G trimers on the virion surface [20,21] can bind cellular receptors, which trigger virion endocytosis and pH-dependent membrane fusion [22,23,24].

**Figure 1:**
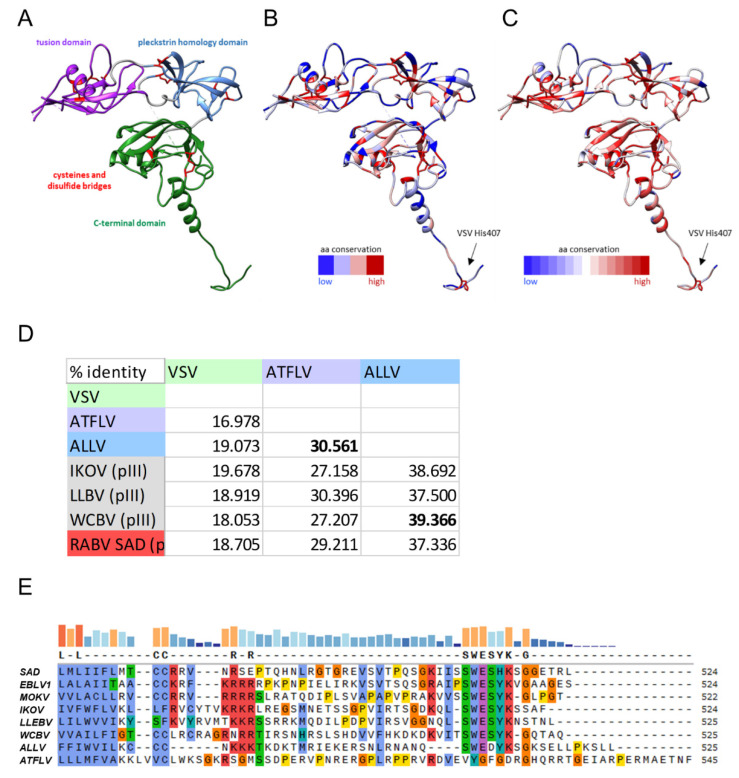
Analysis of G protein sequences. (**A**–**C**) Domain organization and sequence conservation of lyssavirus G proteins. (**A**) Structure of the RABV G protein (pdb: 6LGX) with the structural domains and cysteine residues highlighted. (**B**) Degree of sequence conservation per residue when comparing RABV, MOKV, ALLV, and ATFLV. (**C**) Degree of sequence conservation per residue when comparing the G protein sequences of all known lyssavirus species. The conserved histidine at position 407 of the VSV G protein is highlighted in B and C. The figure was generated with UCSF Chimera. (**D**) Comparison of ATFLV and ALLV G sequence identity with those of phylogroup III lyssaviruses (WCBV-West Caucasian bat lyssavirus; IKOV–Ikoma lyssavirus; LLEBV–Lleida bat lyssavirus) and with RABV SAD (phylogroup I). (**E**) Clustalω comparison of G C-tail sequences. Sequences were ordered according to phylogroups with the novel non-mammalian lyssaviruses added below phylogroup III viruses. Conserved residues are indicated by the colors blue: hydrophobic residues; red: positive charge; magenta: negative charge; green: polar; pink: cysteins; orange: glycines; yellow: prolines; cyan: aromatic residues. Non-conserved residues are black on white. Conservation is indicated by bars on top of the alignment.

Mammalian, frog, and anole lyssavirus G proteins share the same overall organization. All cysteine residues of lyssaviruses are also conserved in ALLV G and ATFLV G, suggesting a similar overall structure and domain folding (Figure 1A). In addition, histidine residue VSV 407, reported to be essential to function as a pH-sensitive switch during fusion [25], is conserved in all lyssavirus G protein sequences. The overall sequence conservation of ALLV and ATFLV G proteins in comparison to RABV and MOKV G is depicted on the structure of RABV G protein (pdb: 6LGX) [26] (Figure 1B) and shows the same pattern of high versus low conserved residues as is observed for an alignment of the G proteins of all lyssavirus species (Figure 1C). This conserved pattern of residue conservation suggests that the same functional limitations govern the sequence variability of the newly discovered reptilian and amphibian lyssavirus G proteins when compared to lyssavirus sequences from mammals.

The ALLV G is more similar in length (525 residues) and sequence conservation to the G proteins of mammalian lyssaviruses. The highest amino acid (aa) identity (39.4%) and identical length is observed with WCBV G, but identities with G proteins of the phylogenetically more distant phylogroup I viruses like RABV SAD B19 G are still comparable (524 aa, 37.3%). ATFLV encodes a longer G protein of 545 aa with a significantly lower sequence conservation of 30.4% identity to that of the most similar virus, LLEBV G, and 29.2% compared to the SAD B19 G protein. With an aa identity of 30.6%, the G proteins of ALLV and ATFLV are approximately equally distinct from each other (Figure 1D). The extra length of ATFLV G is mainly due to an extension of the C-terminal cytoplasmic tail (Figure 1E), which is highly atypical in a sequence compared to all other lyssaviruses. A 6 aa motif conserved throughout mammalian lyssavirus phylogroups (SWESYK), is only slightly modified in ALLV G (SWEDYK), while it is completely lacking in ATFLV G. ATFLV G is thus the most divergent lyssavirus G protein, in accordance with the most proximal position of ATFLV in the phylogeny.

### 3.2. Production of RABVΔG with ALLV G and ATFLV G Envelopes

The function of heterologous viral type I glycoproteins can be analyzed by the phenotypical complementation (namely, pseudotyping) of G-deficient RABV replicons like SADΔG (for a recent review see [27]). While distantly related virus proteins may require engineering of the C-tail for effective RABVΔG pseudotyping [28,29], proteins with a C-tail similar to that of RABV G, e.g., as found in Mokola lyssavirus G [30] can be immediately used for the production of RABVΔG pseudotype particles (RABV pp).

To study whether the novel lyssavirus G sequences are compatible with the RABV pp system, cDNAs of the authentic ALLV and ATFLV G ORF sequences were custom synthesized, expressed from plasmids, and used for the complementation of RABVΔG replicons, specifically SADΔG-eGFP [15], and a newly generated SADΔG-GLuc-mNeongreen reporter replicon. To minimize background infection by the homologous SAD G-complemented RABV pp, we applied the well-established EnvA/TVA system, which is routinely applied in rabies virus monosynaptic tracing experiments, and which involves two sequential envelope switching steps [28]. Stocks of SAD G-complemented RABVΔG were first used to produce EnvA^RT^ pp, which can only infect cells expressing the avian tumor virus A receptor (TVA) [31]. Recombinant HEK293T-TVA cells were then transfected with pcDNA3-ALLV G or -ATFLV G (and control G proteins) and infected with EnvA^RT^ pp to initiate the second envelope switch, which typically yields RABV pp with low RABV G background infectivity for non-avian cells (<10^2^/mL). Any increase in infectivity would indicate the successful incorporation and function of the heterologous G proteins.

**Figure 2:**
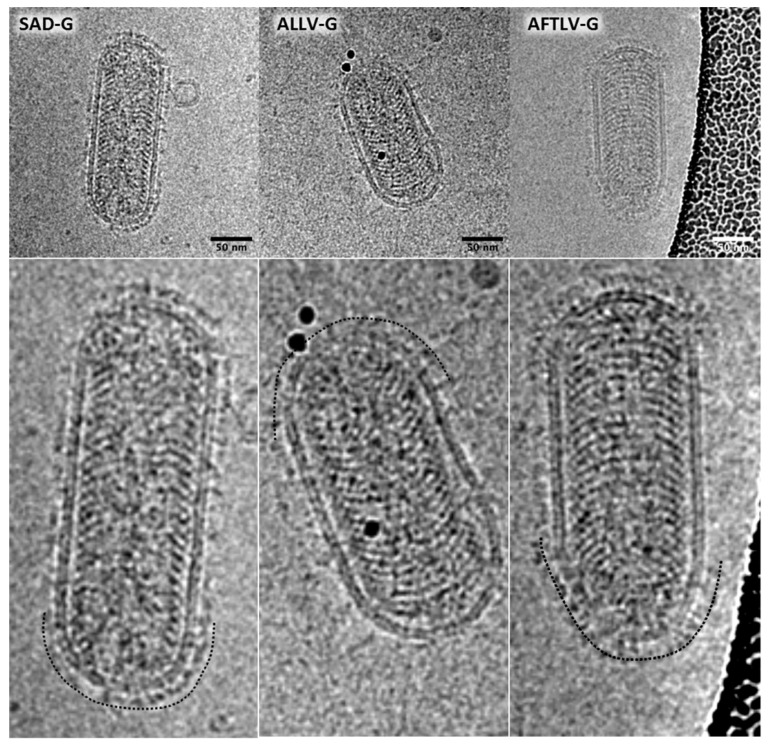
Morphology of ALLV and AFTL G virus particles. RABV pp containing SAD, ALLV, and ATFLV G proteins were analyzed by cryoEM. The original SAD G layer (left) showed a homogenous G protein height of about 8 nm (as indicated by the black checkered line in the detail image), whereas AFTL G protein densities were of a longer and thinner appearance. The coverage of particles with ALLV G protein was poor, and surface glycoprotein densities looked heterogeneous.

Ultracentrifuged RABV pp stocks were analyzed by cryo-EM, which demonstrated the presence of typical rhabdovirus-shaped particles carrying surface proteins in their envelopes (Figure 2). While SAD G-complemented RABV pp revealed a rather uniform homogenous layer of about 8 nm thickness, ATFLV G proteins appeared to be longer and the thickness of the layer more heterogeneous. Although ALLV G was more similar to SAD G in terms of size and C-tail sequence, the coverage of RABV pp with ALLV G proteins appeared to be poor, and of heterogeneous size.

### 3.3. Infection of Human and Rodent Cells

**Figure 3:**
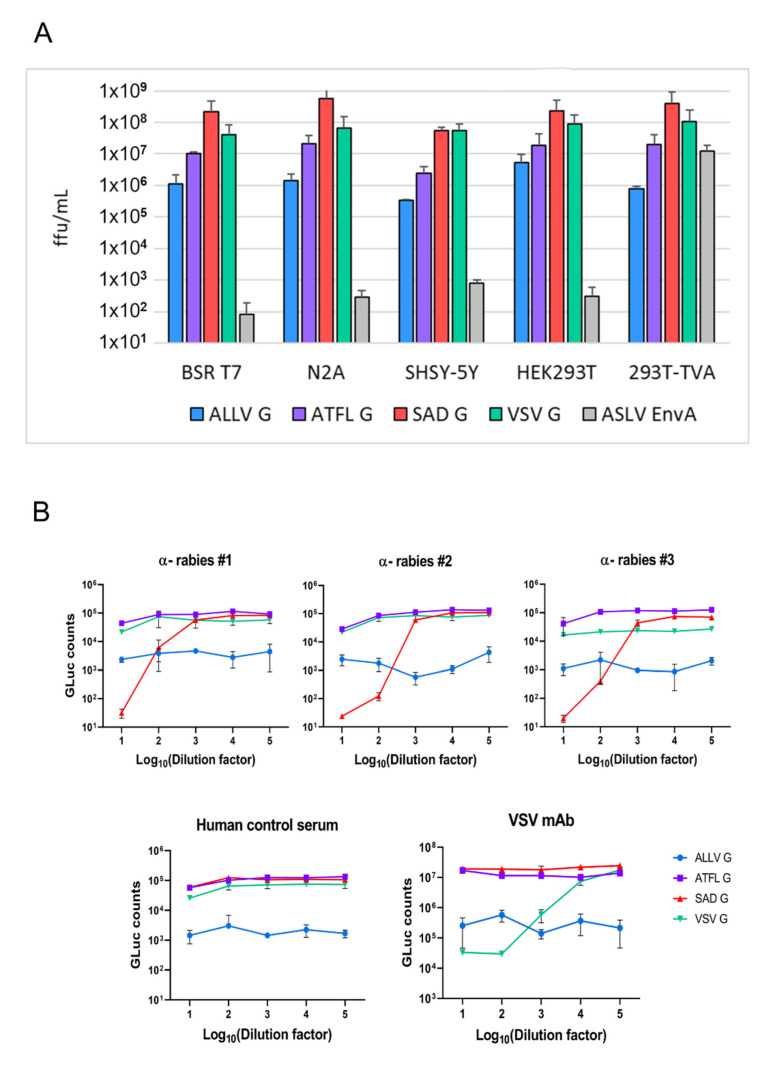
Infectivity of RABV pp for human and rodent cell lines. (**A**) Infectious titers of RABV particles pseudotyped with the indicated G proteins on human and rodent cells, including neuronal (mouse N2A, human SHSY-5Y), and kidney cell lines (human HEK293T, hamster BSR T7/5). The image integrates results from two independent experiments with SADΔG-eGFP and SADΔG-GLuc-mNeongreen replicons and two replicates each. Titers were determined by manually counting eGFP-expressing cells, and the mean of the two distinct replicons and standard deviation are depicted. Titers of the control EnvA pp (input control) were determined in triplicate with SADΔG-GLuc-mNeongreen replicons. (**B**) Specific neutralization of RABV SADG pp by human rabies immune sera and of VSV G pp by a VSV-specific mAb. RABV pp were incubated with serial dilutions of the indicated sera or mAb, and *Gaussia*-Luciferase activity was determined in infected BSR T7/5 cells 3 d.p.i.

To analyze infectivity of the RABV pp for human and rodent human cells, HEK293T and BSR T7/5 kidney cell lines as well as human (SHSY-5Y) and mouse (N2A) neuronal cell lines were used for titration of the pseudotyped viruses. As positive controls, we employed RABV pp complemented with the homologous SAD G and VSV G^RT^, a chimeric VSV G with the CT of RABV SAD G [32] as well as the original EnvA^RT^ pp stock employed for the final envelope switching. The latter confirmed high titers (10^7^/mL) exclusively for TVA expressing cells, while in TVA-negative cells background titers of around 10^2^/mL were determined (Figure 3A).

**Figure 4:**
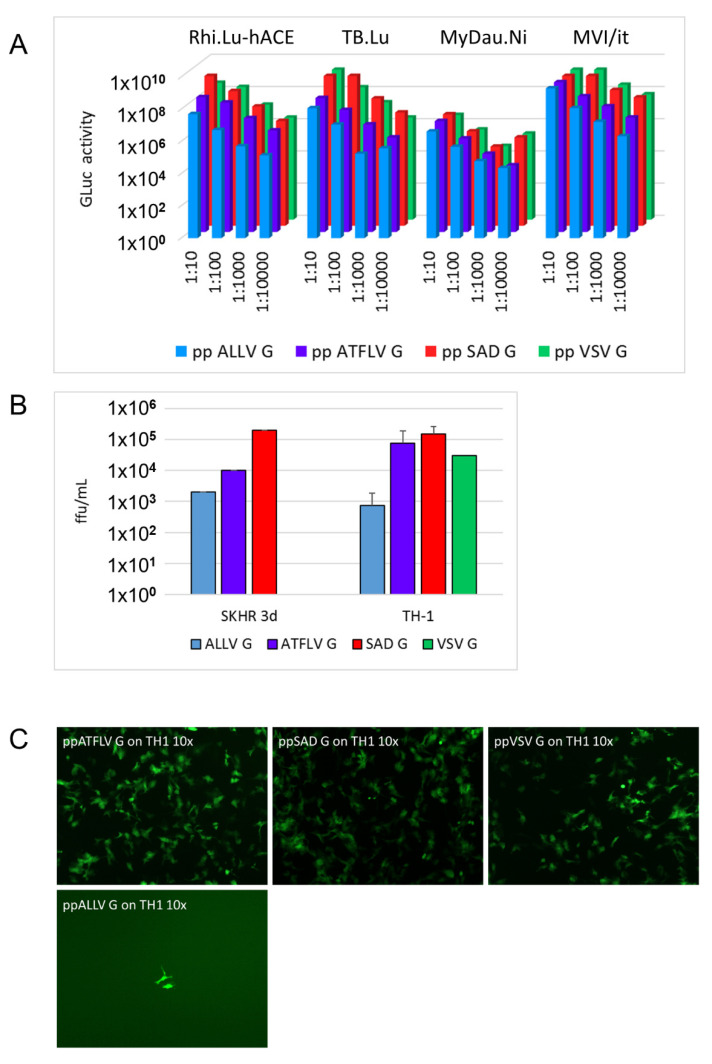
ALLV and ATFLV G pp can infect bat and reptile cells. (**A**) The indicated bat cell cultures were incubated with serial dilutions of RABV pp stocks (SADΔG-GLuc-mNeongreen), and *Gaussia* luciferase activity was determined after 3 d.p.i. (Rhi-Lu-hACE2, *Rhinolophus* sp. lung, expressing hACE2; TB1-Lu, *Tadarida brasiliensis* lung; MVI/it, *Myotis velifer incautus;* MyDauNi/2, *Myotis daubentonii*). Note that reduction of activity of ATFL and ALLV with respect to SAD G RABV pp (red) roughly correlates with physical titers observed in Figure 3. (**B**) Infection of reptile cells. Common box turtle (TH-1) and red-eared slider snake (SKH-R) cell lines were infected with the indicated RABV pp stocks (SADΔG-GLuc-mNeongreen) and titers were determined by counting fluorescent cells 3 d.p.i. Note that ATFL pp reaches titers almost equivalent to SAD G pp. (**C**) Representative micrograph of TH-1 cultures infected with 1:10 dilutions of RABV pp stocks.

Both ATFLV G and ALLV G could effectively mediate the infection of RABV pp in all tested rodent and human cell cultures, illustrating the functional replacement of the SAD G protein. While titers of ATFLV pp were reduced in average by 50-fold compared to SAD G pp, a 500-fold reduction was observed for ALLV G pp, which might reflect the apparently less dense surface coverage of pp as seen in the Cryo-EM images. Importantly, however, the pattern of the susceptibility of cells to infection by the different lyssavirus G pp was similar. Of the neuronal cells, murine N2A cells revealed an approximately ten-fold higher permissivity compared to human SH-SY5Y. Rodent and human kidney cells showed an approximately equal susceptibility. No major difference in cell type and host specificity was observed for VSV G^RT^ pp (Figure 4A).

After having confirmed the infectivity of ALLV G and ATFLV G pp for mammalian cells, the serological cross-reaction was studied. RABV pp expressing *Gaussia* luciferase were incubated with serial dilutions of human rabies immune and control sera before the infection of BSR T7/5 cells and luciferase assays three days later. Rabies immune sera exclusively neutralized the infectivity of SAD G pp at dilutions below 1:1000, while no inhibitory effect was observed on ATFLV-, ALLV-, or VSV G pp (Figure 3B). A VSV mAb (“Ix”) specifically neutralized the infectivity of VSV G^RT^ pp, while the human control serum did not affect the infectivity of any of the RABV pp. These results corroborated the specificity of infection by ATFLV and ALLV G proteins and confirmed their antigenic distance from the phylogroup I lyssavirus G proteins.

Considering the natural host reservoirs of the majority of lyssavirus species, infection studies were extended to include bat cells Rhi-Lu hACE2, TB-1 Lu, MyDauNi/2, and MVI-it. Infection and gene expression was determined by luciferase assays. Cells were infected with a 10-fold dilution series of the above RABV pp stocks, and secreted *Gaussia* luciferase activity was determined after incubation for 3 days. Dose-dependent activity was observed with all pp in all cell types, revealing the successful infection of bat cells (Figure 4A). As was presumed from the above GFP reporter experiments in human and rodent cells, SAD G and VSV G pp yielded the highest GLuc activity in all bat cell lines, while counts for ATFLV and ALLV G pseudotypes revealed reductions roughly reflecting the titer differences observed in cells from terrestrial mammals. In summary, the above results reveal that cell lines from a broad variety of mammalian hosts are permissive for RABV pp carrying reptilian and amphibian lyssavirus G proteins, and with preferences similar to those of rabies SAD G pp.

### 3.4. Infection of Reptile Cells

The rabies virus was reported to infect cells from lower vertebrates and invertebrates [33,34]. This allowed us to include cell lines more closely related to the natural hosts of ALLV and ATFLV. Cell lines of snake (SKH-R; VH-2), lizard (Ig-H), and turtle (TH-2) origin were cultured at 29 °C and a 2.5% CO_2_ atmosphere and were found permissive for entry and gene expression of SAD G RABV pp, although at considerably lower efficiency compared to mammalian cells. The highest titers for SAD G pp were observed in SKH-R and TH-1 cells, hardly reaching 10^6^/mL (Figure 4B). All reptile cell lines were also permissive for ATFLV and ALLV G pp. While lower susceptibilities were again observed for ATFLV and ALLV G pp in SKH-R, the infection of turtle TH-1 and viper VH2 cells with ATFLV G pp indicated titers identical or higher than those of SAD G pp or VSV G pp cells (Figure 4C). Whether the equal uptake of ATFLV G and SAD G pp in these cells is due to an intrinsic advantage of the former or a disadvantage of the latter remains to be determined in detailed future experiments addressing the potential host preferences of anole and frog lyssavirus entry.

## 4. Discussion

A tremendous number of novel viral genome sequences are currently being identified by next generation sequencing (NGS) of genomes and transcriptomes. While until recently, the culturing of a virus in its host and the characterization of multiple phenotypic attributes were required for qualification as a real virus by the International Committee on Taxonomy of Viruses (ICTV), in the age of NGS the genome sequence is now sufficient for proposing a new virus species or taxonomic group [35]. Our results reveal that the original G proteins encoded by the novel frog and anole lyssavirus-like sequences are fully competent in mobilizing genetic material in the form of RABV replicons and mediating infection. These results, along with the finding of high levels of ALLV mRNA transcripts covering the entire genome [11], support the existence of transferable lyssaviruses in poikilothermic animals.

Once we could demonstrate the functional competence of G proteins encoded by anole and frog lyssaviruses to form infectious pseudotype particles, the broad spectrum of permissive host cells in vitro did not come as a big surprise. Rhabdoviruses like VSV or RABV can use ubiquitously expressed and conserved receptors, or a multiplicity of alternative receptors, respectively. While VSV uses members of the conserved LDLR family [36] for attachment, endocytosis, and pH-dependent membrane fusion, divergent proteins can support infection with RABV, including p75NTR, NAChR, NCAM-1, and mGluR2 [37,38,39,40,41,42]. In addition to mammalian cells, ALLV and ATFLV G pp could infect reptile cells, as is known for RABV [33,34]. With respect to a broad host tropism in vitro and the ability to mediate the infection of a diversity of cells from mammals and poikilothermic animals, frog and anole lyssavirus G proteins are therefore highly similar to the RABV G protein.

More unexpected, however, was the finding that ATFLV G, despite its much greater genetic distance to RABV and deviations from the “standard” lyssavirus G protein, including an elongated C-tail lacking an otherwise conserved motif of lyssavirus G proteins, yielded higher RABV pp titers on all cells tested than the more rabies-similar ALLV G. This also applied to cells from reptiles, which initially were presumed to be highly permissive for the reptile ALLV G pp. This observation, however, does not necessarily indicate the lower intrinsic infectivity or different receptor usage of the latter protein, but may be associated with the reduced incorporation in RABV envelopes, as was suggested by the Cryo-EM analysis. Both reduced compatibility with SADΔG particles and the kinetics of protein processing and transport in HEK293T cells could contribute to the lower infectious titers obtained under the standard conditions used here. Further quantitative experiments comparing identically tagged protein versions in different cell types may shed light on the potential differences in the processing and infectivity of G proteins.

Importantly though, the cell, organ, and host tropism of lyssaviruses in vivo are not exclusively governed by cell entry, but also by specific restrictions thwarting virus propagation. In the case of RABV, the host innate immune system may profoundly shape the virus–host relationship [43,44,45]. Intriguingly, both amphibian and reptilian lyssavirus-like sequences were identified in brain samples [11], suggesting they share the typical in vivo neurotropism of all lyssaviruses. Future screening and surveillance might reveal whether they also share their capability of causing lethal encephalitis. Since a natural spillover from bat lyssaviruses to terrestrial animals and humans has been reported, though as sporadic events [46], it is important to experimentally clarify whether the novel viruses pose a threat to heterologous hosts.

Animals other than mammals or birds are usually not considered immediate candidate reservoirs for zoonotic viral infections, or even the sources of epidemics and pandemics [2]; however, snakes were brought forward as intermediate hosts in the case of SARS-CoV-2, because of a similar synonymous codon usage bias [47]. In any case, in vivo experiments with lyssaviruses require due diligence, particularly in view of the antigenic differences and insensitivity to rabies vaccination-induced neutralizing antibodies (Figure 3B). Single round virus systems as employed here and previously [6,27,48] enable safe and informative approaches, such that the potentially hazardous generation of full-length chimeric lyssaviruses [49] appears to not be immediately necessary.

While it will be of interest for experimental virologists to learn more about the biology of the novel ATFLV and ALLV, including details on G structure, replication, host immune interplay, and pathogenesis in mammalian and poikilothermic hosts, the finding of lyssaviruses in poikilothermic animals challenges the current view of lyssavirus evolution. Genetic and antigenic distances exclude recent cross-species transmission events and argue in favor of a common ancestor of vertebrate lyssaviruses. In fact, recent studies suggest that the phylogenetics of many mammalian-associated virus taxa have to be reconsidered as they were found to be also present in reptiles, amphibians, or fish hosts, and many of them shared tissue tropism with their mammalian counterparts [50,51]. Likewise, members of the *Lyssavirus* genus seem be more widespread, and the genus older than currently appreciated.

## Data Availability

All data are presented in the manuscript.
